# Propagation of CJD Prions in Primary Murine Glia Cells Expressing Human PrP^c^

**DOI:** 10.3390/pathogens10081060

**Published:** 2021-08-20

**Authors:** Joo-Hee Wälzlein, Karla A. Schwenke, Michael Beekes

**Affiliations:** Prion and Prionoid Research Unit, ZBS 6—Proteomics and Spectroscopy, ZBS—Centre for Biological Threats and Special Pathogens, Robert Koch Institute, Nordufer 20, 13353 Berlin, Germany; SchwenkeK@rki.de (K.A.S.); BeekesM@rki.de (M.B.)

**Keywords:** prions, Creutzfeldt–Jakob disease, human cell model, glia

## Abstract

There are various existing cell models for the propagation of animal prions. However, in vitro propagation of human prions has been a long-standing challenge. This study presents the establishment of a long-term primary murine glia culture expressing the human prion protein homozygous for methionine at codon 129, which allows in vitro propagation of Creutzfeldt–Jakob disease (CJD) prions (variant CJD (vCJD) and sporadic CJD (sCJD) type MM2). Prion propagation could be detected by Western blotting of pathological proteinase K-resistant prion protein (PrP^Sc^) from 120 days post exposure. The accumulation of PrP^Sc^ could be intensified by adding a cationic lipid mixture to the infectious brain homogenate at the time of infection. Stable propagation of human prions in a long-term murine glia cell culture represents a new tool for future drug development and for mechanistic studies in the field of human prion biology. In addition, our cell model can reduce the need for bioassays with human prions and thereby contributes to further implementation of the 3R principles aiming at replacement, reduction and refinement of animal experiments.

## 1. Introduction

Prion diseases are a group of progressive and always fatal neurodegenerative disorders also known as transmissible spongiform encephalopathies (TSEs). They include Creutzfeldt–Jakob disease (CJD) and its variant form (vCJD) in humans, scrapie in sheep and goats, bovine spongiform encephalopathy (BSE) in cattle and chronic wasting disease (CWD) in cervids. Clinical manifestation of TSEs is characterised by a variety of cognitive and moto-sensory symptoms including dementia, cognitive decline, impaired speech, incoordination, etc. [1]. According to the prion hypothesis, TSEs are caused and transmitted by proteinaceous infectious particles, prions, composed of misfolded and pathologically aggregated host-encoded prion protein (PrP). While the normal cellular form of PrP is referred to as PrP^C^, its TSE-associated pathological isoform is usually designated as PrP^Sc^ [2] or PrP^TSE^ [3]. The conversion of PrP^C^ into its aggregated isoform PrP^Sc^ can be triggered spontaneously, genetically or by infection with prions.

For the last several decades, prion research has been largely dependent on bioassays and model prions from laboratory animals, which is not least due to the fact that the development of cell models for human prion propagation has proven to be extremely challenging. Thus, animal experiments are often still considered indispensable for drug development, studies on prion biology and the biological detection and titration of TSE agents [4,5]. However, increasing efforts have recently been made to implement the principle of the three Rs (replace, reduce and refine) in order to alleviate and curb animal studies. Increasingly stringent and restrictive regulations on animal experiments and ethical as well as cost issues emphasise the need for robust and easy-to-access cell models for human prion propagation.

While neurons are the primary target cells for prion replication, it has been shown that other cell types, especially astrocytes and other glia cells, also play important roles in prion biology and are in fact among the earliest sites of prion accumulation. It was further shown that intercellular connections involving astrocytes contain PrP^Sc^ and possibly mediate its spread from cell to cell via tunnelling nanotubes. The intercellular transfer of aggregated prion protein by astrocytes may have an impact on disease progression [6]. Thus, apart from neuronal cultures, an additional focus has been put on glia cells when it comes to developing cell models for prion propagation [7,8,9,10]. The few existing cell models which already show human prion propagation [9,11,12], involve labour-, cost- and time-intensive techniques, making them generally difficult to access. Cell models propagating human prions are especially desirable in order to investigate the molecular mechanisms underlying human prion disease and, potentially even more important, to conduct drug development as well as prion decontamination studies. The latter has lately been of increased interest since other neurodegenerative diseases (e.g., Parkinson’s and Alzheimer’s disease (PD and AD)) have come into the spotlight with their emerging prion-like properties in terms of self-templated propagation of disease-associated protein aggregates [13,14].

In this report, we present a novel cell assay based on primary glia cell cultures prepared from transgenic mice expressing human PrP homozygous for methionine at codon 129 [15], which is easily accessible and allows robust in vitro propagation of human prions (vCJD and sCJD type MM2).

## 2. Results

### 2.1. Establishment of a Long-Term Primary huMM129 Mixed Glia Culture

Isolation and cultivation of primary murine glia from transgenic mice expressing human PrP homozygous for methionine at codon 129 of the prion protein gene (huMM129) were carried out as described previously [7] with modifications. The main adjustment to the isolation protocol was a two-step enzymatic digestion of the brain tissue rather than just mechanic disintegration. This resulted in a cell culture mainly consisting of astrocytes [16], which represent a vital cell type of prion propagation in vitro. Cells were isolated from huMM129 pups on postnatal day 1–3 (P1–3) and cultures were maintained in a healthy state for up to 150 days without passaging.

### 2.2. Propagation of sCJD-MM2 and vCJD Prions 

**Figure 1:**
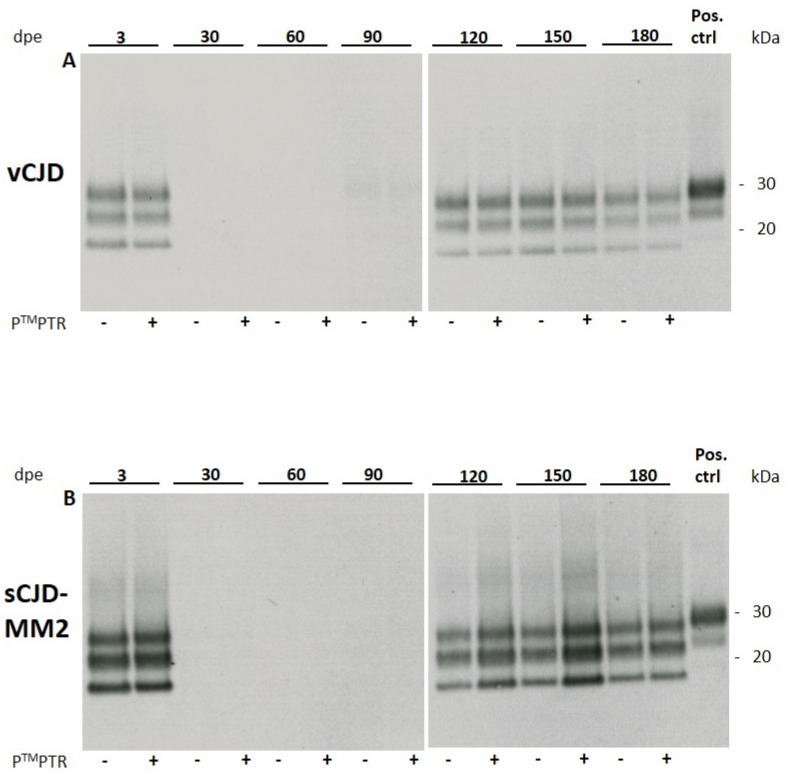
Western blot analysis of cell lysates of huMM129 glia infected with vCJD (**A**) or sCJD-MM2 prions (**B**). 3 × 10^4^ cells/well were challenged with 10 µL of 1% brain homogenate (*w*/*v*) for 3 days. Cells were lysed at 3 days post exposure (3 dpe) and at the indicated time points. Empty lanes at 30, 60 and 90 dpe indicate that no original inoculum was present any more in the glia cell culture. From 120 dpe onwards, propagation of non-residual PrP^Sc^ can be detected. Addition of Pierce^TM^ Protein Transfection Reagent (P^TM^PTR) to the original inoculum results in enhanced propagation of sCJD-MM2 prions but shows no effect for vCJD prions (n = 2; representative Western blots shown). At 180 dpe, this effect disappears again. Positive control: 10% homogenate (*w*/*v*) of 263K-infected hamster brain, diluted 1:1000. Anti-PrP antibody 3F4 used at 1:2000.

HuMM129 cultures were exposed to sCJD-subtypes MM1 and MM2 and vCJD prions at 1:10 and 1:100 dilutions of a 10% brain homogenate (*w*/*v*) as previously described for 263K hamster scrapie prions [7]. While sCJD-MM1 prions did not replicate in vitro to detectable levels, sCJD-MM2 and vCJD prions showed in vitro propagation at a 1:10 dilution, which became detectable at 120 days post exposure (120 dpe) by Western blotting for PrP^Sc^ (Figure 1). The glycosylation pattern of the non-residual PrP^Sc^ signal did not show any noticeable alteration compared to the signal of the original inoculum, which leads to the assumption that no obvious changes in strain properties occurred during prion propagation in our glia cultures.

In cells which were exposed to a 1:100 dilution of infectious brain homogenate (10% *w*/*v*), prion replication did not reach detectable levels. Prion propagation was consistently observed in two independently produced huMM129 glia cell preparations and two different samples of both sCJD-MM2 and vCJD homogenate.

#### 2.2.1. Pierce^TM^ Protein Transfection Reagent Enhances Prion Propagation in huMM129 Glia Cultures for sCJD-MM2 but Not for vCJD Prions

Addition of the cationic lipid mixture Pierce^TM^ Protein Transfection Reagent (P^TM^PTR; Thermo Fisher, USA) resulted in an apparently stronger PrP^Sc^ signal in glia cells challenged with sCJD-MM2 prions at 120 dpe compared to cells which did not receive P^TM^PTR. The lipid mixture did not have any effect on the extent of vCJD propagation (Figure 1). P^TM^PTR was not able to stimulate propagation of sCJD-MM1 prions to detectable levels (Table 1).

#### 2.2.2. Deterioration of Cell Viability Starting at 150 dpe Results in Declining PrP^Sc^ Signal

From about 150 dpe, glia cultures started to deteriorate and showed reduced cell viability. Viability was assessed by visual evaluation of cells (not shown). Cells showed increasing detachment from the culture vessel and displayed an atypical round morphology. Due to the decrease in healthy cells susceptible to prion replication, which was observed from 150 dpe, the signal intensity of PrP^Sc^ stagnated and subsequently declined. At 180 dpe, the P^TM^PTR-induced increase in PrP^Sc^ signal intensity was less pronounced compared with the signals seen at 120 and 150 dpe.

## 3. Discussion

### 3.1. Low Proliferation Rate of Glia Cells Allows for PrP^Sc^ Accumulation

It has previously been reported that (fast) proliferating cells are not able to propagate human prions [9]. This is presumably caused by the fact that human prions replicate very slowly in vitro and constant cell division results in the dilution of accumulated PrP^Sc^ to non-detectable levels. One of the cell models shown to propagate human prions exploits postmitotic astrocytes [9], emphasising the plausibility of cell division hindering PrP^Sc^ accumulation. Our cell model is based on mixed glia cultures containing mainly astrocytes and, to a smaller degree, microglia, according to findings in similarly prepared cell cultures from hamsters and bank voles [7,16,17]. Mature astrocytes are considered to be proliferation quiescent [18,19], which is in agreement with the fact that we were able to maintain our glia cultures in six-well plates for at least 150 days without passaging. We strongly suggest that non-proliferating cultures are highly advantageous for the successful propagation of human prions. This presumption is further supported by previous studies in our lab, which showed that exposure of human immortalised astrocytes [20] to sCJD or vCJD prions did not result in detectable levels of PrP^Sc^ (data not shown). Those cells are highly proliferative and require passaging at least twice a week. PrP^Sc^ accumulation might possibly have occurred in these human immortalised astrocytes but was diluted by frequent cell passaging to non-detectable levels.

We can only speculate as to why we observed prion propagation for sCJD-MM2 and vCJD but not for sCJD-MM1 prions. One obvious explanation might be that sCJD-MM1 and -MM2 as well as vCJD prions comprise different protein conformations, which differently favour or hinder PrP^Sc^ replication in our cell cultures. Furthermore, we observed a relatively strong cytotoxic effect of sCJD-MM1 prions during the first three days following infection. This initial cytotoxicity possibly resulted in a substantially reduced cell number of viable cells throughout the course of the assay, and therefore the level of PrP^Sc^ propagation remained below level of detection. Visual assessment did not disclose a massively reduced cell density in the affected wells, but since we did not evaluate culture properties with viability or cytotoxicity assays, we cannot rule out this possibility.

### 3.2. Alteration of Cell Membrane Permeability May Increase Effectiveness of Prion Infection In Vitro

A common transfection method is the reversal of membrane polarity in order to increase membrane permeability by cationic lipids. It has been shown that the cationic lipid Pierce^TM^ Protein Transfection Reagent (P^TM^PTR, Thermo Fisher, Waltham, MA, USA) significantly increases the infectivity of RML prions in the scrapie cell assay [21]. Based on this finding, we utilised this reagent in our primary glia cell model, resulting in an enhanced PrP^Sc^ signal of sCJD-MM2 prions from 120 dpe compared to non-P^TM^PTR-treated cells. The variably reduced number of viable cells observed from 180 dpe onwards apparently diminished the enhanced propagation of sCJD-MM2 prions induced by P^TM^PTR. The effects seen at 120 and 150 dpe may have been caused by coating infectious prions in a positively charged layer of amphipathic molecules, therefore facilitating uptake into the negatively charged cell membrane [22]. This was, however, a pilot study and reproducibility needs to be statistically proven. Nevertheless, our findings are an obvious hint that targeting membrane polarity or other physical or chemical measures of increasing membrane permeability could potentially be a very helpful approach to improving the effectiveness of prion infection in vitro. Prior to our infection assays with human prions, we saw the described supportive effect of P^TM^PTR in hamster glia cultures infected with 263 K scrapie prions (*n* = 4; not shown) and are therefore confident that the observed effect is not only due to random variation.An explanation as to why we observed enhanced prion propagation for sCJD-MM2 but not for vCJD prions could be that vCJD shows a higher infectivity and attack rate in bioassays compared to sCJD, and that prion propagation in vitro might already occur at a relatively high level, which leaves little room for enhancement (by, e.g., P^TM^PTR) as opposed to sCJD prions.

### 3.3. Transferability of the Glia Cell Model to Other Species of Cell Donors and Prion Strains

The simplicity of our cell model in terms of isolation technique and cultivation conditions predestines it to be set up with glia cells of other species, including bank voles, which are considered to be universal acceptors of prion strains [23]. Transgenic mice (over-)expressing prion protein from bank voles, humans or other mammalian species or PrP knockout mice prospectively provide further versatile sources for additional cell models for human or animal prions. The transferability and robustness of the glia cell infection model, originally reported for hamster 263K scrapie and other prion strains in hamster cells [7,24], are further substantiated by the fact that it was recently established in our lab for various mouse-adapted scrapie prions in bank vole cells [16]. Especially in contrast to induced pluripotent stem (iPS) cell-based infection models, which hold a greater potential for mimicking human disease, our glia cell model offers a straightforward, labour- and cost-friendly tool for human prion propagation.

Once established for a broader spectrum of human prion strains and possibly further optimised in terms of infection speed and sensitivity, this cell model may substantially facilitate studies on the pathobiology of human prions and drug therapy of CJD.

## 4. Materials and Methods

### 4.1. Animals and Human Tissue Samples

#### 4.1.1. huMM129 Mice

Cells were sourced from neonatal mice, which are null for mouse PrP but express human PrP homozygous for methionine at codon 129 of the prion protein gene (huMM129, [15]). These mice have a clinically unaffected phenotype.

Euthanasia of huMM129 mice aged 1–3 days for the preparation of brain tissue (which did not require approval for animal experiments) was reported to and registered by the local animal protection authority (“Landesamt für Gesundheit und Soziales Berlin”, Berlin, Germany; registration number T0197/15). All animals used in this study were handled under the European directive regarding the protection of animals used for scientific purposes, in strict accordance with the German Animal Welfare Act (Tierschutzgesetz).

#### 4.1.2. sCJD-MM2 and vCJD Brain Tissue

The work described in this study has been carried out in accordance with The Code of Ethics of the World Medical Association (Declaration of Helsinki) for experiments involving humans. Sampling and use of tissue from vCJD and sCJD patients for scientific purposes were carried out with the understanding and written consent of each donor or his or her authorised caregiver. Use of the tissue for experimental studies and scientific reports such as our work was approved for sCJD specimens by the Ethics Committee of the University Medical Center Göttingen (No. 11/11/93) and for vCJD specimens by the East of Scotland Research Ethics Service (No. 16/ES/0084).

### 4.2. Isolation and Cultivation of Primary Murine Glia Cells

Isolation and cultivation of primary murine glia cells were carried out as previously described [7] with modifications adapted from the Kettenmann lab (Max Delbrück Center, Berlin, Germany). Briefly, brains from neonatal huMM129 mice (postnatal day 1–3, P1–3) were collected. Cerebellum, olfactory bulb and meninges were removed and the remaining tissue was washed three times with ice-cold Hank’s balanced salt solution (HBSS). Enzymatic tissue digestion was achieved in two steps: first by trypsin (10 mg/mL; Biochrom, Berlin, Germany) and DNAseI (0.5 mg/mL; Roche, Basel, Switzerland), incubated at room temperature (RT) for 2 min, followed by DNAseI only (5 mg/mL) with gentle trituration by pipetting. The tissue suspension was centrifuged at 120× *g* for 10 min; the pellet was resuspended in DMEM containing 10% fetal calf serum (FCS; Pan Biotech, Aidenbach, Germany), 1% GlutaMAX and 100 U/mL penicillin/streptomycin (all Thermo Fisher; referred to as DMEM complete); and cells were plated on poly-L-lysine-coated (PLL; 0.01%; Sigma-Aldrich, St. Louis, MO, USA) T75 flasks while allocating three brains per flask. Cells were maintained in a 5% CO_2_ incubator at 37 °C. After two days, cells were washed vigorously four times with PBS and kept in culture for another two to three days. Cells were then split 1:4 and subconfluent cultures were either cryopreserved as described previously [24] or directly used for infection experiments.

### 4.3. Infection of huMM129 Glia Cells with sCJD-MM1/-MM2 and vCJD Prions

HuMM129 glia cells were plated in PLL-coated (0.01%) 6-well plates at 3 × 10^4^ cells/well. sCJD-MM1/-MM2 and vCJD 10% homogenates (*w*/*v*) were sonicated at 300 W for 30 sec in a microplate horn sonicator (Misonix 3000) prior to cell infection.

The 10% brain homogenates were diluted 1:10 and 1:100 in DMEM complete and cells were exposed to 10 µL of 1% and 0.1% homogenates in 2 mL culture medium for three days. Cells were then washed once with PBS, to remove residual homogenate from the cultures, and supplemented with fresh medium. Cultures were maintained for 30, 60, 90, 120, 150 and 180 dpe while supplementing them once a week with fresh medium. In order to prove reproducibility, two independent cell isolations of huMM129 glia were used as well as two different aliquots of both sCJD and vCJD homogenates. Samples for Western blot analysis were taken for each time point; one additional sample was taken at 3 dpe in order to indicate the presence of residual PrP^Sc^ from the original inoculum.

### 4.4. Infection of huMM129 Glia Cells with sCJD-MM1/-MM2 or vCJD Prions in the Presence of Pierce^TM^ Protein Transfection Reagent

It has been shown that prion infectivity can be enhanced in the presence of the cationic lipid mixture P^TM^PTR (Thermo Fisher; [21]). To test whether this effect can be seen in our in vitro cell model, P^TM^PTR was added to the cells at the time of infection with sCJD-MM1/-MM2 or vCJD brain homogenates according to the manufacturer’s instructions. For each time point, cells were infected with CJD prions with and without addition of P^TM^PTR.

### 4.5. Western Blot Analysis

Western blot analysis for the detection of accumulated PrP^Sc^ was performed as previously described [4,6] with modifications. Cells were washed once with PBS and harvested in 100 µL of 1% sarcosyl (*v*/*v*). In total, 10 µL of the cell lysate was digested with 75 µg/mL proteinase K (PK; Roche) at 37 °C for 1 h and further denatured in 2× sample loading buffer at 110 °C for 10 min. Ten percent hamster brain homogenate (*w*/*v*) of a 263K scrapie-infected animal diluted 1:1000 served as a positive control. SDS-PAGE electrophoresis was performed on BOLT 4–12% Bis-Tris mini protein gels, and proteins were blotted onto PVDF membranes using the iBlot 2 dry blotting system (Thermo Fisher). PVDF membranes were probed with anti-PrP monoclonal antibody 3F4 (1:2000; inhouse production) at 4 °C overnight and anti-mouse IgG alkaline phosphatase-linked secondary antibody (1:5000; Dako, Santa Clara, CA, USA). Protein bands were detected with CDP-Star chemiluminescent substrate (Thermo Fisher) for alkaline phosphatase chemiluminescence reaction on Amersham Hyperfilm^TM^ ECL films (GE Healthcare, Chicago, IL, USA), which were exposed for 40 min.

## Figures and Tables

**Table 1:** Prion replication in huMM129 primary glia detectable from 120 dpe and PrP^Sc^ signal enhancement by P^TM^PTR.

	sCJD-MM1	sCJD-MM2	vCJD
Prion propagation Detectable at 120 dpe (or later)	no	yes	yes
Signal enhanced by P^TM^PTR	no	yes	no

## Data Availability

The data produced in these experiments are provided by the corresponding author on request.
